# Supplementary material for: Conformational Defects in the Limbs of Menorca Purebred Horses and Their Relationship to Functionality
Source: Animals (Basel). 2024 Mar 31;14(7):1071. doi: 10.3390/ani14071071 (PMC11011047; doi:10.3390/ani14071071)
Supplement: Supplementary file 1 [file animals-14-01071-s001.zip › animals-2924794-supplementary.pdf]

Supplementary table S1. Description of the 14 limb conformation defects analyzed in the Menorca Purebred Horse population, including the definition of the evaluation scale.

| Defect | Class | | Defect | Class | |
|---|---|---|---|---|---|
| | 0 (correct) | 2 (evident) | | 0 (correct) | 2 (evident) |
| Open hock *OH* | Correct angle of hock in lateral view[2] | Very open angle of hock in lateral view | Closed hock *CH* | Correct angle of hock in lateral view[2] | Very closed angle of hock in lateral view |
| Convergent *Conv* | Correct direction of hock in rear view[2] | Very convergent direction of hock in rear view | Divergent *Diver* | Correct direction of hock in rear view[2] | Very divergent direction of hock in rear view |
| Camped under *CU* | Correct angle of forelimb in lateral view[1] | Significant under deviation of the angle of forelimb in lateral view | Camped out *CO* | Correct angle of forelimb in lateral view[1] | Significant outside deviation of the angle of forelimb in lateral view |
| Pigeon-toed forelimb *PTF* | Correct fore-hooves stance of forelimb[1] | Significant inside deviation of hooves stance of forelimb | Splay-footed forelimb *SFF* | Correct fore-hooves stance of forelimb[1] | Significant outside deviation of hooves stance of forelimb |
| Base narrow *BN* | Correct pastern and foot stance of forelimb[1] | Significant inside deviation of pastern and foot stance of forelimb | Base wide *BW* | Correct pastern and foot stance of forelimb[1] | Significant outside deviation of pastern and foot stance of forelimb |
| Coon foot *CF* | Correct hoof-pastern axis of forelimb[1] | Broken hoof-pastern axis of forelimb with horizontal tendency | Broken and upright foot *BUF* | Correct hoof-pastern axis of forelimb[1] | Broken hoof-pastern axis of forelimb with vertical tendency |
| Sloping foot *Sloping* | Correct Fore fetlock joint angle[1] | Broken hoof-pastern axis of forelimb with horizontal pastern | Club foot *Straight* | Correct Fore fetlock joint angle[1] | Broken hoof-pastern axis of forelimb with upright pastern |

[1] The hind limb conformation can be considered correct when a plum line dropped from point of buttocks touches rear border of the hock, run parallel to cannon and strike the ground 3-4 inches behind the heel. From the rear, the line should bisect hock, cannon, pastern and heel. [31]

[2] The forelimb conformation can be considered correct when the forearm ties right into center of knee. The knee is flat and facing straight ahead. The cannon bone comes from center of knee and extends to center of fetlock. The pastern comes from center of fetlock and drive into center of hoof. [31]
